# Predicting the relationship between pesticide genotoxicity and breast cancer risk in South Indian women in *in vitro* and *in vivo* experiments

**DOI:** 10.1038/s41598-023-35552-3

**Published:** 2023-06-15

**Authors:** S. Sasikala, M. Minu Jenifer, K. Velavan, M. Sakthivel, R. Sivasamy, E. R. Fenwick Antony

**Affiliations:** 1 Molecular Genetics and Cancer Biology Laboratory, Department of Human Genetics and Molecular Biology, Bharathiar University, Coimbatore, Tamil Nadu 641 046 India; 2 Erode Cancer Center Hospital, Perundurai Road, Thindal, Erode, Tamil Nadu 638012 India; 3 Department of Statistics, Bharathiar University, Coimbatore, Tamil Nadu 641 046 India

**Keywords:** Cancer, Genetics, Molecular biology, Environmental sciences

## Abstract

Breast cancer is the third most common cancer in women after skin and lung cancer. Pesticides are of interest in etiologic studies of breast cancer because many pesticides mimic estrogen, a known breast cancer risk factor. In this study, we discerned the toxic role of the pesticides atrazine, dichlorvos, and endosulfan in inducing breast cancer. Various experimental studies, such as biochemical profiling of pesticide-exposed blood samples, comet assays, karyotyping analysis, pesticide and DNA interaction analysis by molecular docking, DNA cleavage, and cell viability assays, have been carried out. Biochemical profiling showed an increased level of blood sugar, WBC, hemoglobin, and blood urea in the patient exposed to pesticides for more than 15 years. The comet assay for DNA damage performed on patients exposed to pesticides and pesticide-treated blood samples revealed more DNA damage at the 50 ng concentration of all three pesticides. Karyotyping analysis showed enlargements in the heterochromatin region and 14pstk+, and 15pstk+in the exposed groups. In molecular docking analysis, atrazine had the highest glide score (− 5.936) and glide energy (− 28.690), which reveals relatively high binding capability with the DNA duplex. The DNA cleavage activity results showed that atrazine caused higher DNA cleavage than the other two pesticides. Cell viability was the lowest at 50 ng/ml (72 h). Statistical analysis performed using SPSS software unveiled a positive correlation (< 0.05) between pesticide exposure and breast cancer. Our findings support attempts to minimize pesticide exposure.

## Introduction

Breast cancer (BC) is the most common type of cancer among women worldwide, and it is the fifth most common cause of death according to the World Health Organization (WHO)^1^. Breast cancer causes the largest number of cancer-related deaths among women with malignancies in the developed and developing world. Types of BCs include ductal, lobular, invasive ductal, invasive lobular, phyllodes tumor, tubular carcinoma, mucinous carcinoma, and medullary cancer^2^. According to reports of the National Cancer Registry Program of the Indian Council of Medical Research (ICMR), approximately thirteen thousand Indians die every day from cancer. Over time, various material resources were made available to treat cancer but they were shown to work for very few subjects, and many fail to respond to such treatments^3^. Medical investigation of breast cancer in the absence of proper methods for early detection is still challenging. On the other hand, changes in health and lifestyle patterns in the lifetime might be the cause of cancer. In the United States, one out of every eight women has breast cancer in their lifetime. Researchers have reported that the average age of women with cancer ranges between 45 and 59^4^. Reports for the involvement of environmental exposures such as certain chemicals, diet and social factors in breast cancer progression were exciting with evidence. Endogenous and exogenous substances such as pesticides are continuously subjected to damage to human cells^5^. A number of these substances structurally disrupt DNA and can change or stop essential cellular functions such as DNA replication or transcription^6^. Base and sugar alterations, single- and double-strand breaks, DNA‒protein cross-links, and base-free sites are among the most frequently occurring DNA damage processes. Cells have evolved a specific DNA repair system that may be separated into various pathways depending on the type of DNA damage to overcome the negative consequences of DNA damage. Important macromolecules, including lipids, proteins, and DNA, are damaged when oxidative stress and reactive oxygen species (ROS) are produced in excess^7^. Chromosomal abnormalities (CAs) are one of the important biological impacts of ionizing radiation and other environmental pollutants (pesticides) on human health. The prevalence of CA in peripheral blood lymphocytes has been linked to a significantly increased risk of cancer development in women^8^. The frequency of structural chromosome aberrations is regarded as a sensitive predictor of mutagenic carcinogen exposure, which may even cause chromosomal anomalies, DNA damage, and abnormal signal transduction followed by genotoxicity, and new findings show that those with high chromosome aberration frequencies have a higher cancer incidence. In silico docking can explore the biochemical mechanisms of ligand–protein interactions. Over the past 30 years, unstable aberrations and acentric fragments have been detected by chromosomal aberration testing, such as karyotyping. These tests have been able to quantify the extent of genomic instability caused by physical and chemical agents^9,10^. Most cancer genomes undergo large modifications that profoundly alter their structure and content. This characteristic of genomic instability is the root cause of a wide variety of chromosomal aberrations found in cancer genomes. With the available information on chromosomal aberrations in cancer, the identification of significant aberrations has become more precise and effective.

### Genotoxic effects of Atrazine, Endosulfan and Dichlorvos

**Figure 1:**
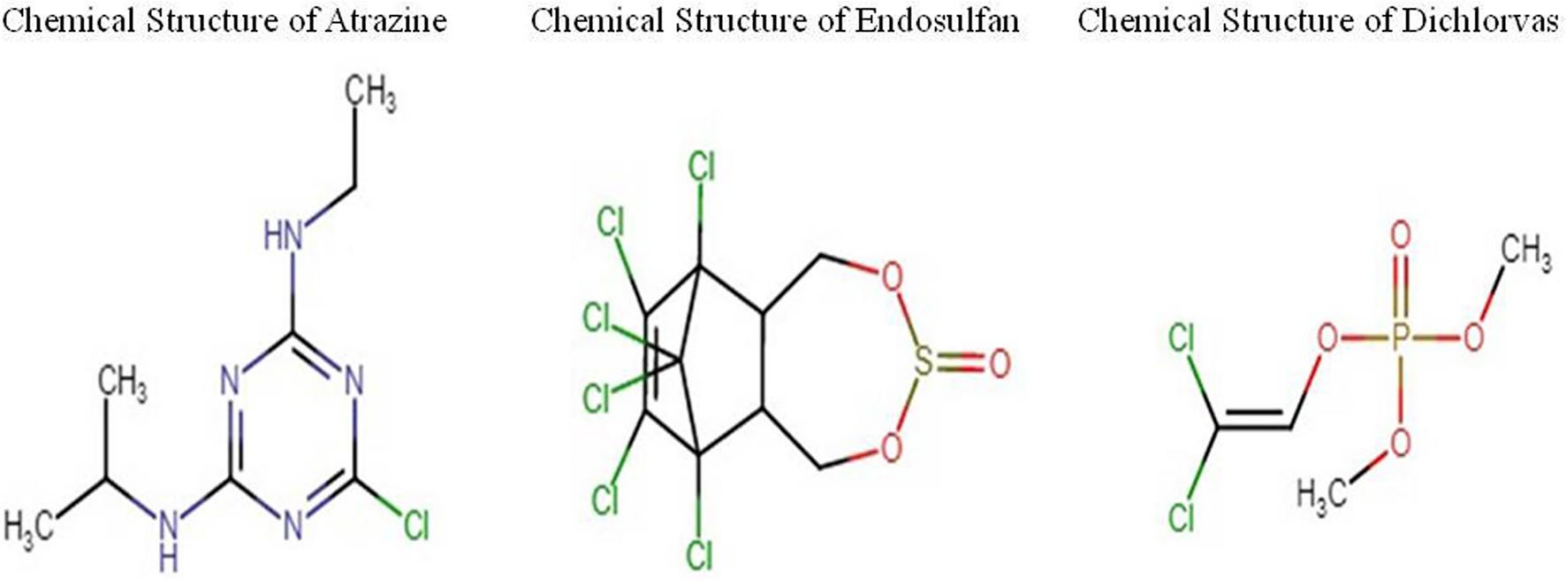
Pesticide chemical structure.

Atrazine (Fig. 1) is an organochlorine pesticide that belongs to the chloro-s-triazine family of herbicides. Atrazine has been broadly utilized in farmlands and regularly identified in rural watersheds, which are considered a potential risk to human wellbeing^11^. There are nearly 300 products that contain atrazine. Products with atrazine may be labeled for use on soil, roadsides, lawns, and athletic fields. Certain products can be used on corn, sorghum, sugarcane, macadamia nuts, guava, or wheat stubble after harvest. After exposure to atrazine, it can remain on human skin for 24 h, and only 6% of atrazine is absorbed by our body. Atrazine is well absorbed orally by our body and enters the gastrointestinal tract. As it moves through the body, atrazine may be found in the liver, ovary, kidney, red blood cells, and fat. Atrazine biotransformation is achieved through a series of phases. Atrazine biotransformation in humans has not been completely characterized, whereas in rodents, it has been proven that atrazine conjugates with glutathione and causes atrazine mercapturate formation and N-dealkylation. Atrazine is excreted from urine in the form of atrazine mercapturate, which constitutes the major portion of urinary metabolites. Excretion of atrazine from urine usually occurs by approximately 1–2 days after exposure and through feces 2–4 days after exposure. When rats were exposed to a 95% dose of atrazine, all its byproducts were excreted within 7 days. As per the USA Environmental Protection Act 1994, it has been reported that feeding atrazine to rats caused an increase in benign and malignant mammary gland tumors in females. Feeding atrazine to male rats can cause an increased incidence of typically rare benign mammary tumors^12^. Atrazine has been reported to cause an earlier onset and increased incidence of mammary gland tumors in female Sprague‒Dawley rats. Experimental studies performed through the comet assay and chromosomal aberration analysis indicated that atrazine may cause carcinogenesis by damaging the integrity of DNA and the stability of the cell genome^13^. According to the U.S. EPA, as published in 1994, dichlorvos is an organophosphate insecticide used in greenhouses, fruits, vegetable crops, and livestock and as a fumigant. Feeding dichlorvos to rats caused an increase in mammary gland tumors in females. Dichlorvos (Fig. 1) exerts its toxic effects in humans and animals by inhibiting neural acetylcholinesterase activity^14^. It was reported that the dose level of Dichlorvos on Chinese hamster ovary (CHO) cell line results showed that the number of mitotic cells harvested was insufficient for a significant SCE analysis at a dose > 1 mg/ml due to its cytotoxic effect. There is also a case report stating that two pesticide workers in Costa Rica died after spilling a dichlorvos containing insecticide on their skin without washing it off properly^15,16^. An experimental study on pesticide exposure to dichlorvos reported that microglial cells undergo cell death after 48 h of dichlorvos treatment. In another study, nigrostriatal neuronal death was reported following chronic exposure to 2.5 mg/day dichlorvos in rats. A case report study was conducted on the effect of induced dichlorvos in a 49-year-old Chinese woman causing autoimmune hepatitis following chronic exposure to dichlorvos. The diagnosis was made two and a half years after the initial symptoms of exposure^17^. Another case report was that of a 19-month-old girl who died following ingestion of a cake-like bait that contained dichlorvos^18^. The carcinogenic roles of endosulfan have been validated by several research experiments. Endosulfan is estrogenic as determined by laboratory tests (causes breast cancer cells to increase in number) and promotes the formation of the “bad” estrogen linked to an increased risk of breast cancer. Endosulfan (Fig. 1) is the fifth most commonly detected pesticide on U.S. produce and is used strikingly on a wide variety of fiber, fruit, and vegetable crops. It was shown that humans exposed to endosulfan show adverse reproductive defects such as reduced male fertility, neonatal deaths, and congenital birth defects. Endosulfan has adverse endocrine-disrupting effects on humans and has been found to evoke estrogenic responses in human breast cells by mimicking estrogen and interfering with the normal levels of estrogen receptors. It has been shown to cause proliferation, transformation, differentiation and migration of human estrogen-sensitive breast cancer cells and estrogen-sensitive ovarian cells^19^. Endosulfan has been shown to have acute oral and inhalation toxicity, as well as slight dermal toxicity. This compound is an irritant to the eyes and is not a dermal sensitizer. Endosulfan is neither mutagenic nor carcinogenic. Endosulfan primarily affects the nervous system. Toxic effects were observed in animals that were found to have acute, sub chronic, developmental neurotoxicity, and chronic/carcinogenic toxicity, and studies found that endosulfan causes neurotoxic effects, which were believed to result from overstimulation of the central nervous system^20^. Furthermore, there is evidence (effects observed in a submitted chronic oral toxicity study in rats) that endosulfan acts as an endocrine disruptor. However, further investigation is necessary to determine the relevance and impact of such findings on public health.

## Results

### Biochemical profile

**Figure 2:**
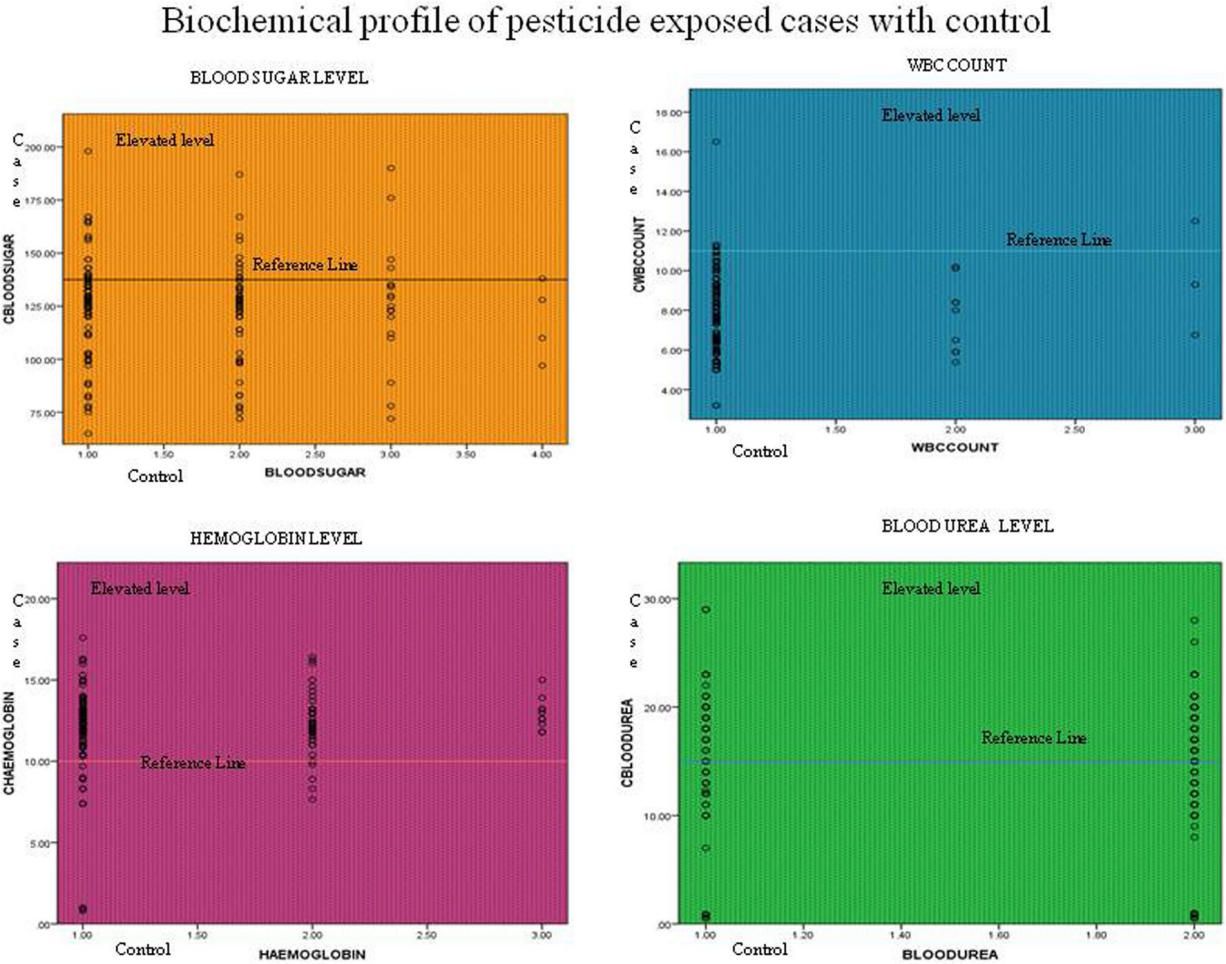
Elevated biochemical profile of pesticide-exposed cases compared with controls. (Elevated levels of blood sugar, blood urea, hemoglobin and WBC count are highlighted in the graph).

The study findings regarding patient blood sugar levels were divided into four categories: (i) normal, (ii) below normal, (iii) prediabetes, and (iv) diabetes. A total of 56.5% of individuals had normal blood sugar levels, 32.2% had blood sugar levels below normal, only 9.9% had blood sugar levels indicative of prediabetes, and 1.5% had diabetes. Low WBC counts occur in cancer patients. The findings of our study showed that, in comparison to their control group (0.9%), 34.6% of subjects had WBC counts that were below normal. A normal hemoglobin level range can identify pesticide metabolites or residues to validate acute exposures. The average hemoglobin level from the study revealed that those who had been exposed to pesticides for more than 15 years had a significant probability of having hemoglobin levels below 7.9, while 15.1% of subjects had moderate hemoglobin levels compared to their control hemoglobin levels. The study found that groups who had been exposed to pesticides had higher mean urea levels than controls. Approximately 70.5% of subjects had abnormal levels of blood urea. Breast cancer cases were analyzed for selected parameters, blood sugar, WBC, hemoglobin, and blood urea, which were significant at the 5% level. Overall, elevated total blood sugar, WBC, hemoglobin, and blood urea levels were highly significant in women with breast cancer compared with the control group. The median result and *p* value of blood sugar, WBC count, hemoglobin, and blood urea have a *p* value of 0.001, which is significant. Table 1 shows data on elevated levels of blood sugar, white blood cells, hemoglobin, and blood urea in pesticide-exposed subjects. These increased levels may play an important role in carcinogenesis (Ref. Table 1, Fig. 2).

**Table 1:** Biochemical Profile of Pesticide Exposed cases with controls.

Parameters	Case	Control	*P* value
Blood sugar	124. 46 ± 167*	1. 5735 ± 122. 89	0.000*
WBC count	7.8546 ± 6.78108*	1.0735	0.000*
Hemoglobin	12. 2875 ± 7*	1. 3235 ± 10. 9639	0.000*
Blood urea	15.6142 ± 14.065*	20	0.000*
Platelet count	2.9505	3.0634 ± .0.11289	0.099
Serum creatinine	0.7368 ± 113.426	0.9314	0.167

*Values significant at 0.05 levels.

### Comet assay

**Figure 3:**
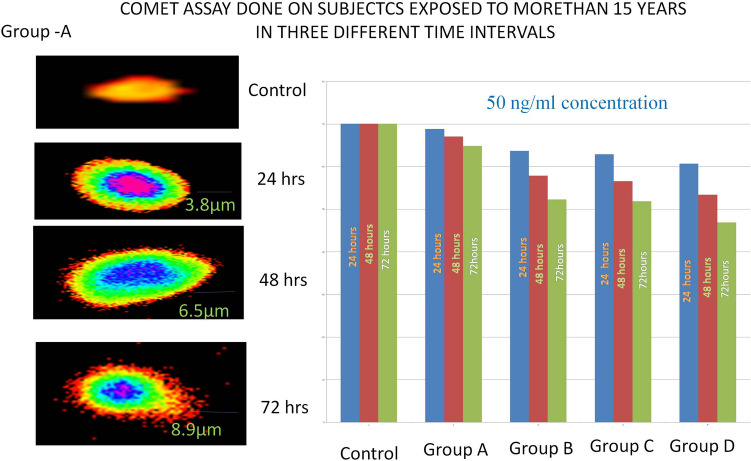
Comet assay. Comet assay performed on subjects exposed to more than 15 years in three different time intervals.

**Figure 4:**
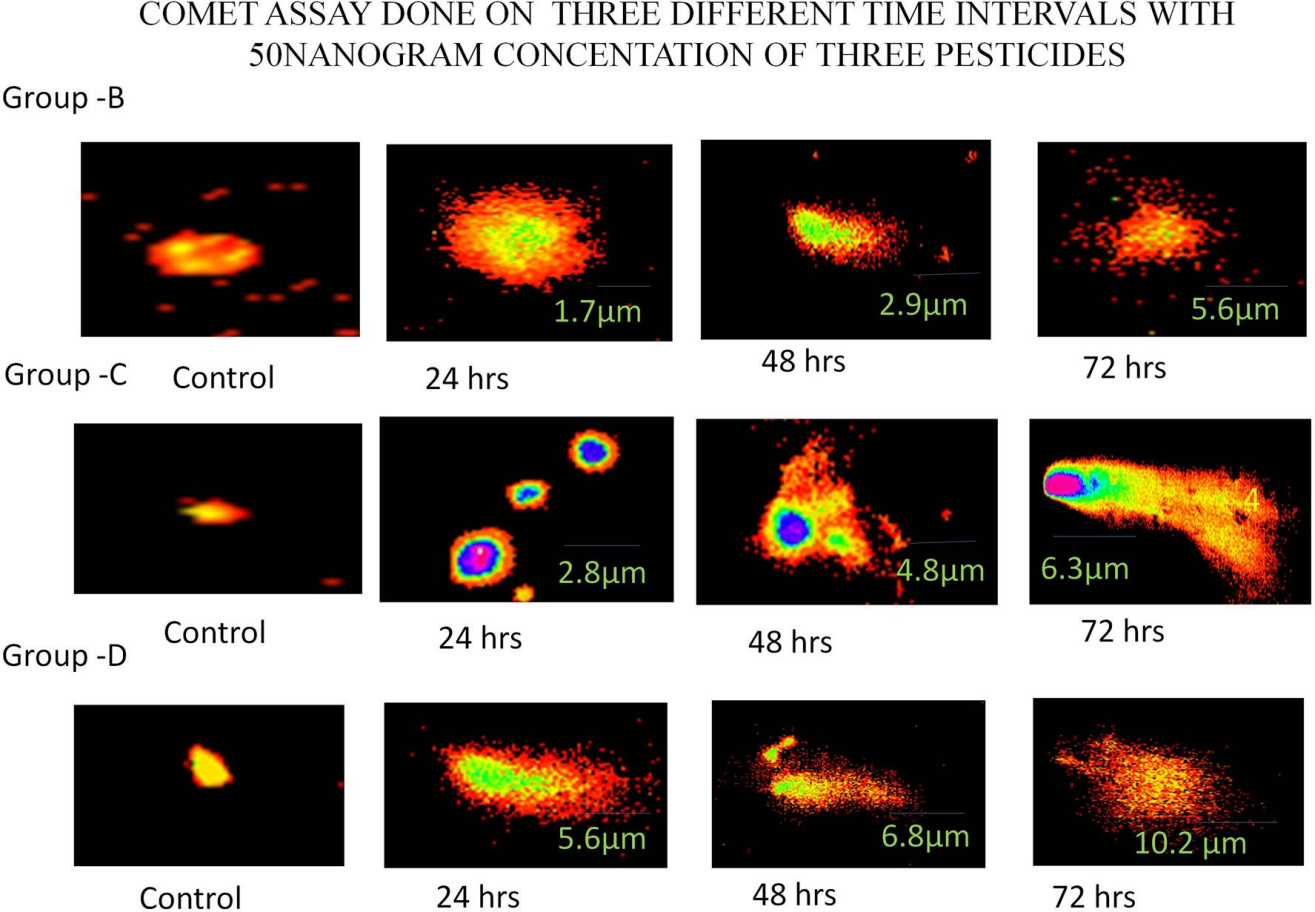
Comet assay performed at three different time intervals with 50 ng/ml concentrations of three pesticides.

Fifteen cells from 50 randomly captured comets per sample from each slide were examined using comet score 2.0 software. These comets were observed at 400X magnification using a fluorescence microscope (Olympus). The comet assay was performed in four groups. (Group A—samples obtained from patients exposed to pesticides for more than 15 years, Groups B, C and D—blood samples treated with various concentrations of atrazine, dichlorvos, and endosulfan, respectively (10–50 ng/ml). A slide with no DNA damage was used as a control. The results showed that DNA strand breakage was detected in Group A. The DNA breaks were quantified by measuring the tail length and the percentage of DNA in the tail. Group A showed a significant mean tail length and percentage of tail DNA value in three intervals: 24 h—285.56 ± 6.39, 48 h—272.38 ± 3.95, and 72 h—289.23 ± 3.14. The DNA strand breaks were observed in Group B (atrazine treatment) with a mean value of tail length and percentage of tail DNA in the 24-h pesticide-treated samples of 55.73 ± 0.89, a value of 56.27 ± 1.23 in the 48-h samples, and a value of 55.83 ± 1.21 in the 72-h treated samples. The DNA damage in three consecutive intervals showed a varied degree of DNA damage, with the highest DNA damage occurring at the 50 ng/ml concentration. Group C blood samples treated with dichlorvos pesticide showed higher DNA damage at the 50 ng/ml concentration than at lower concentrations. The mean value of tail length and percentage of tail DNA was calculated in three intervals: 24 h—190.99 ± 5.88, 48 h—195.70 ± 2.90, and 72 h—189.69 ± 3.63. Group D (endosulfan treated) showed higher DNA damage at the 50 ng/ml concentration than at lower concentrations. The mean values of tail length and percentage of tail DNA were calculated as 4.99 ± 1.85 at 24 h, 4.55 ± 1.57 at 48 h and 4.06 ± 1.73 at 72 h (Ref. Figures 3 and 4 and Table 2).

**Table 2:** Farmers and treatment individuals' comet assay findings and frequency ratios were compared.

Parameters	Group A Patients exposed pesticides for more than 15 years (ng/ml)	Group B healthy controls treated with 50nanogram Atrazine Pesticides (ng/ml)	Group C healthy controls treated with 50nanogram Dichorvas Pesticides (ng/ml)	Group D healthy controls treated with 50nanogram Endosulpan Pesticides (ng/ml)
Control	174.34 ± 2.66	55.62 ± 0.82	87.94 ± 1.84	3.62 ± 0.92
24 h	285.56 ± 6.39*	55.73 ± 0.89	190.99 ± 5.88*	4.99 ± 1.85
48 h	272.38 ± 3.95*	56.27 ± 1.23	195.70 ± 2.90*	4.55 ± 1.57
72 h	289.23 ± 3.14*	55.83 ± 1.21	189.69 ± 3.63*	4.06 ± 1.73

*Values significant at 0.05 levels.

### Quantification of DNA breaks

$${\text{Tail}}\,{\text{moment}} = {\text{tail}}\,{\text{length}} \times \% \,{\text{of}}\,{\text{the}}\,{\text{DNA}}\,{\text{in}}\,{\text{the}}\,{\text{tail}}$$

The results obtained from the comet assay clearly showed the DNA damage occurring in relation to exposure to pesticides. The pesticides were administered at varying concentrations from 10 to 50 ng to determine the maximum concentration at which the DNA was damaged. The experiment was split into four groups; the control was performed with a normal blood sample where no DNA damage was observed. Group A included subjects who were exposed to pesticides for more than 15 years. Group A showed maximum DNA damage with regard to pesticide exposure, and DNA damage was determined based on the tail length and percentage of DNA in the tail. Groups B, C and D were healthy blood samples taken and treated with three highly toxic pesticides, atrazine in Group B, dichlorvos in Group C, and endosulfan in Group D, and all the groups were checked for DNA damage at three time intervals: 24 h, 48 h, and 72 h. The above results clearly indicated that this high concentration of pesticides produced severe DNA damage in the peripheral blood lymphocyte cells. After the three time intervals, 50% of cells produced comets and large tail lengths and tail moments, unlike the negative controls.

### Karyotyping analysis

**Figure 5:**
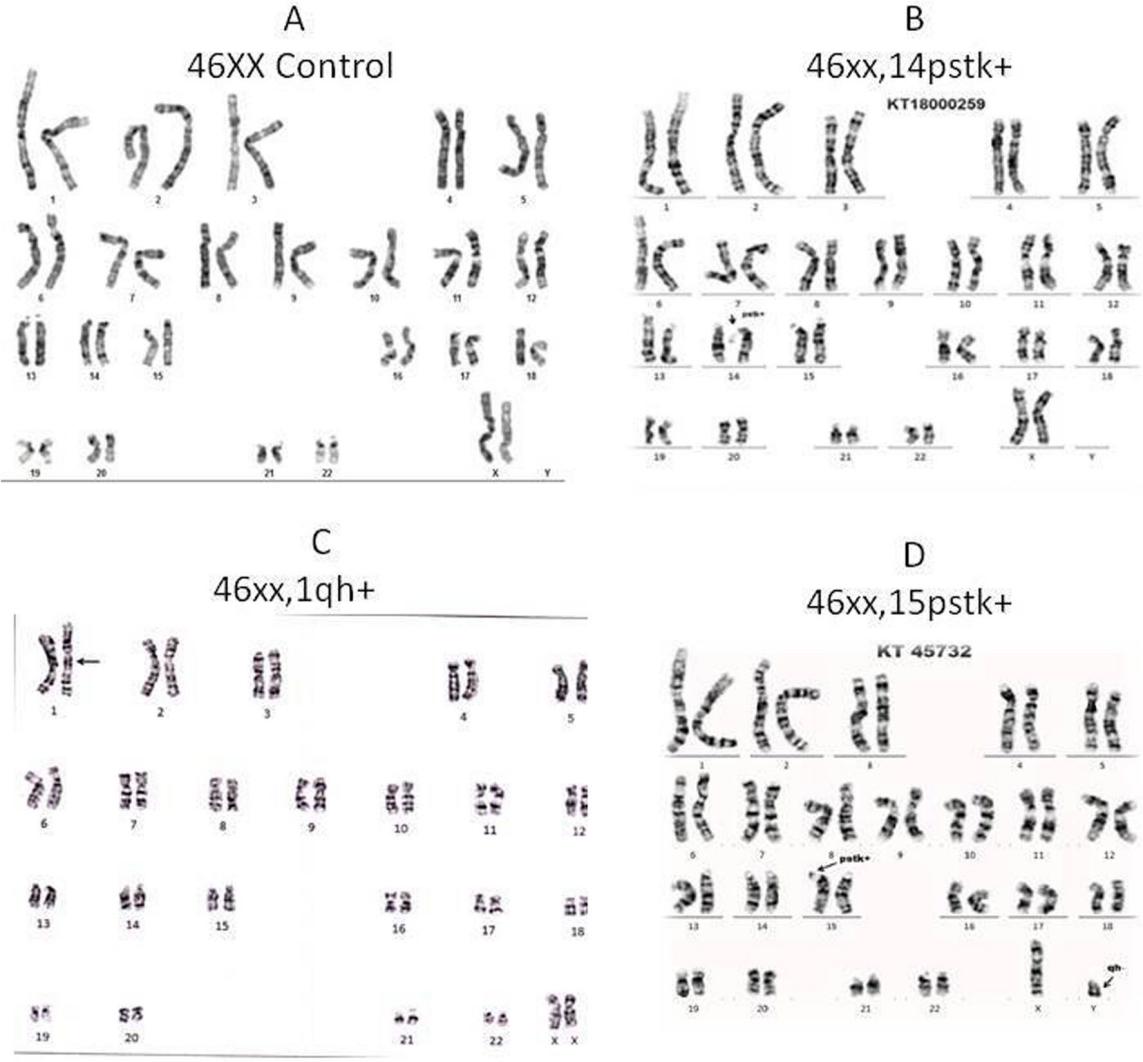
Chromosomal aberration assays. (Subjects exposed to more than 15 years and their aberrations 14pstk+, 15pstk+Satellite and 1qh+Heterochromatin region enlargements because of pesticide exposure).

Karyotyping analysis showed the most prevalent structural changes involved in chromosomes 1, 14 and 15. The results confirmed the gains of the 14pstk+, 15pstk+ satellite and 1qh+ heterochromatin regions, which have been reported to be involved in the early steps of cancer progression. The combination of gains in 1qh+, 14pstk+, and 15pstk+satellites is a frequent cytogenetic abnormality that has been described in up to 30–40% of all breast cancer cases. In addition, the combination of cytogenetic alterations occurring in environmentally induced cancers, especially cancers that occur due to pesticide exposure, has been scientifically proven in earlier studies. The results clearly show that patients who had been exposed to pesticides for more than 15 years were at high risk for breast cancer. Furthermore, several breast cancer patients showed satellite region 14pstk+, 15pstk+ and 1qh+ heterochromatin region enlargements that were found in pesticide-exposed groups, and there were no obvious chromosomal abnormalities in controls. It was observed that those with longer pesticide exposures had higher chromosomal aberrations. The chromosomal abnormalities in the meta-phase are shown in Fig. 5. The results of chromosomal aberrations seen among breast cancer patients were calculated, and their mean SD results showed a 0.05% significant value, as described in Table 3.

**Table 3:** Chromosomal abnormalities in people who have been exposed to pesticides for more than 15 years.

Chromosomal aberrations	Mean ± SD (CA)
Controls	0. 31 ± 0. 48*
1qh +	3. 7 ± 0. 5*
14pstk +	5. 31 ± 0. 95*
15pstk +	9. 1 ± 2. 01*

*Values significant at 0.05 levels.

### Molecular docking

**Figure 6:**
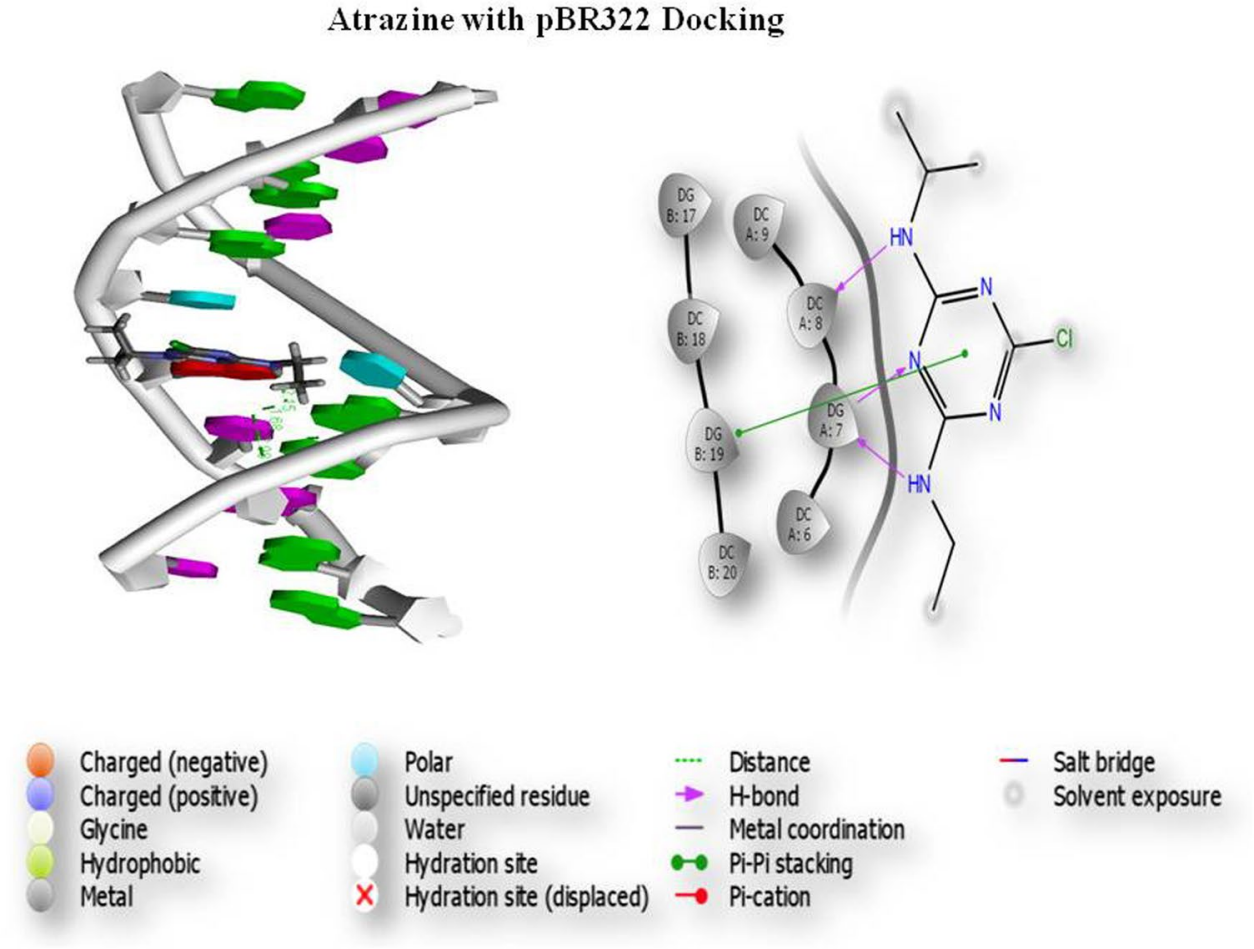
Atrazine and pBR322 DNA docking analysis.

**Figure 7:**
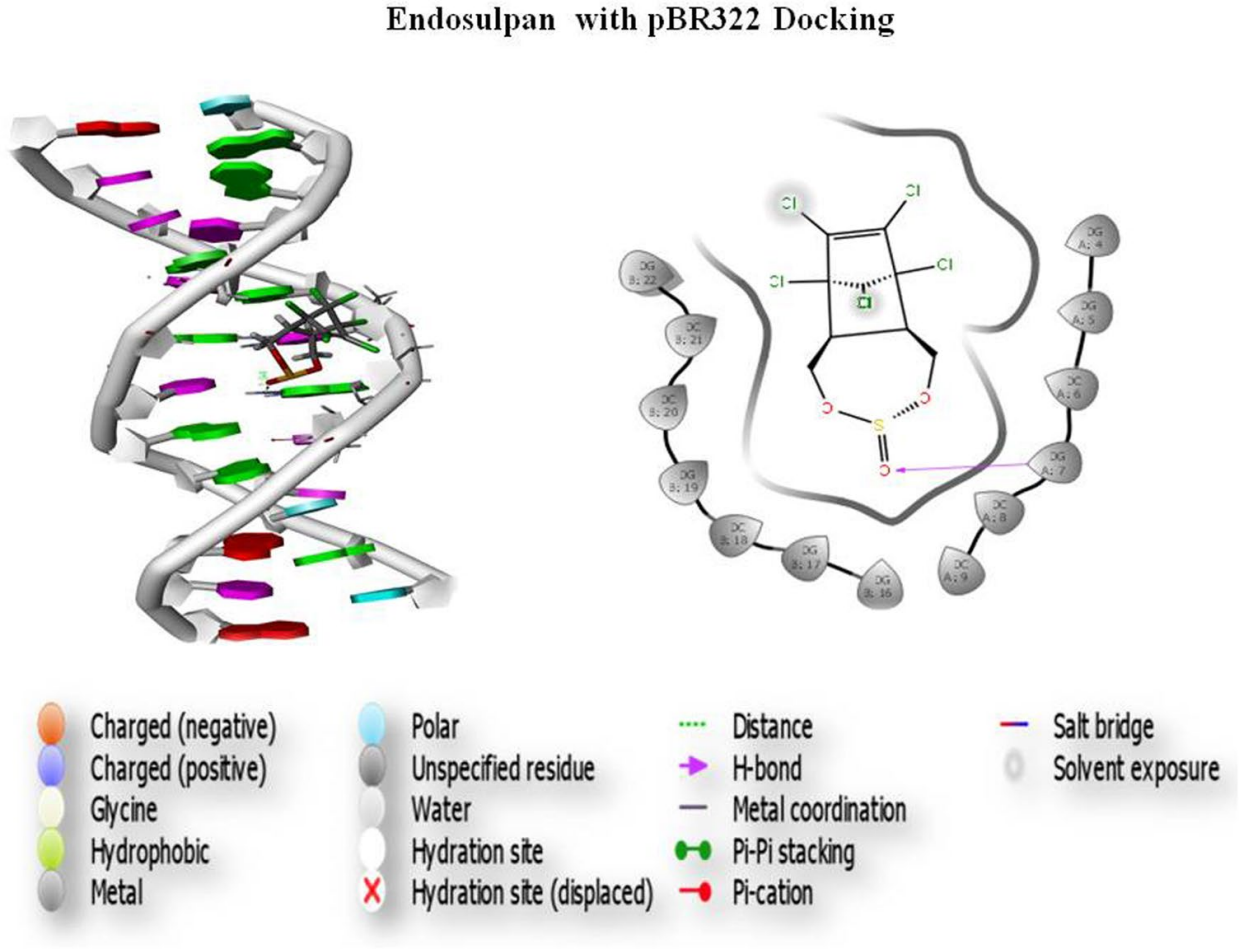
Dichlorvos and pBR322 DNA docking analysis.

**Figure 8:**
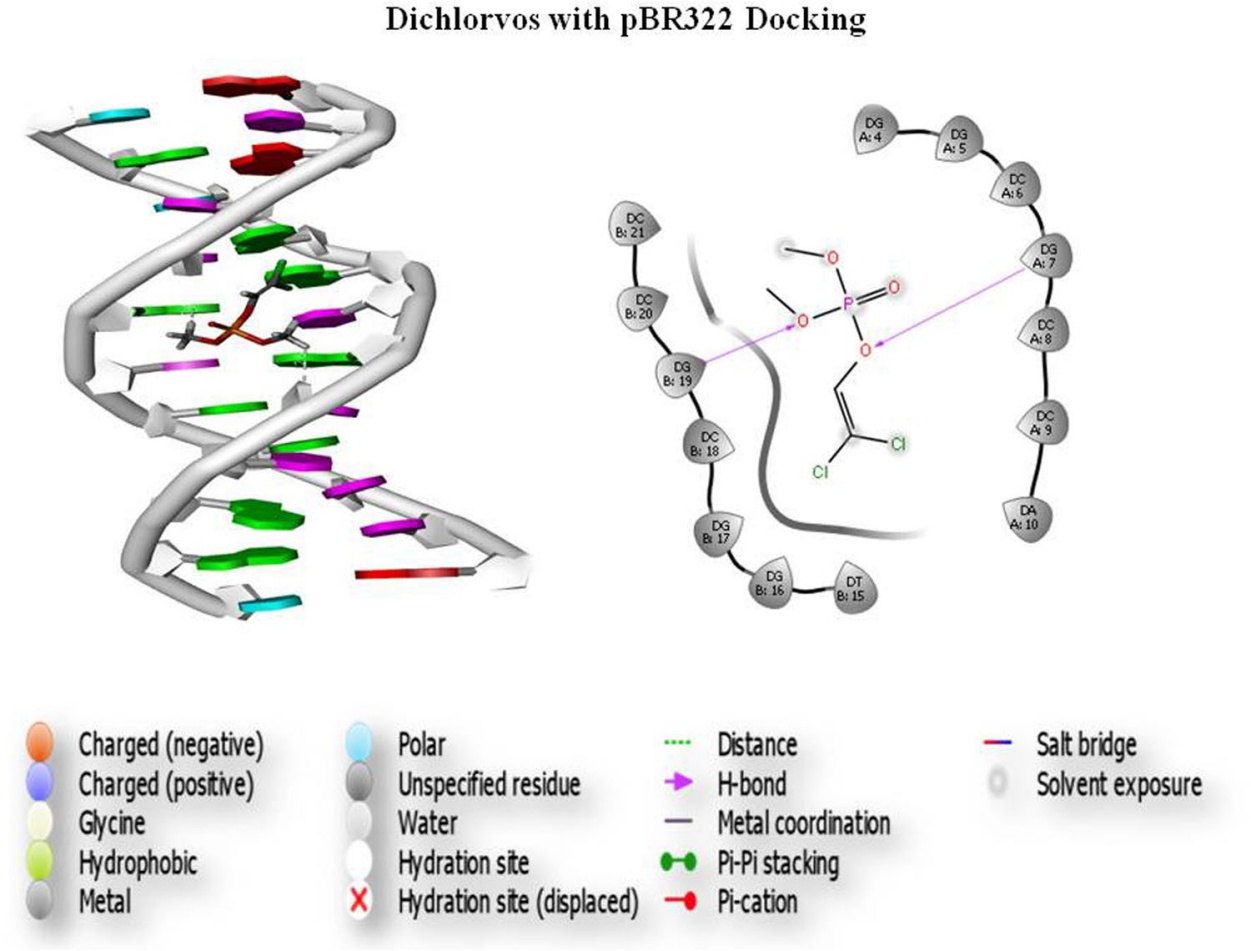
Endosulfan and pBR322 DNA docking analysis.

Gene toxicity analysis was performed by a group of pesticides, and thepBR322 DNA was docked. Three screened compounds were subjected to docking of pesticide and DNA interaction analysis. Initially, based on the scoring function, all the selected compounds were screened. The results of glide docking of the molecules with the DNA (5'-D (*AP*CP*CP*GP*GP*CP*GP*CP*CP*AP*CP*A) -3') for the A chain and DNA (5'-D (*TP*GP*TP*GP*GP*CP*GP*CP*CP*GP*GP*T) -3') for the B chain length 12 are presented in Fig. 1. Glide docking demonstrates that the best pose of Compound 1 (atrazine) has a better glide score than the other 2 compounds. The glide score in the best poses of Compound 1 was − 5.936 kcal/mol. The best docking pose of the compound atrazine showed that the glide energy was − 28.690 kcal/Mol (Fig. 6). This demonstrates that the compound atrazine recognizes both strands of the DNA and binds within the grooves of the helix. Hydrogen bonding indicates that the compound atrazine involves the interaction of 7, 8, and 19 bases of the DNA in DGA, DCA and DGB, respectively. Molecular docking results showed that this compound recognizes both the strands of the DNA (5'-D(*AP*CP*CP*GP*GP*CP*GP*CP*CP*AP*CP*A) -3') for the A chain and DNA (5'-D (*TP*GP*TP*GP*GP*CP*GP*CP*CP*GP*GP*T) -3') for the B chain within the minor groove. The docking results reveal that Compound 1 has the best binding capability with the DNA duplex. The results indicate that the binding of the drug molecules to DNA is sequence-dependent and that the specific sequence of the DNA may play a key role in the binding process. These results may provide insight for future drug development and toxicity assessment. The glide score of atrazine was − 5.936, and the glide energy was − 28.690; atrazine exhibited a good glide score and potentially interacted with pBR322 DNA. This interaction formed 2 hydrogen bonds interacting with 7, 8, and 19 bases in in DGA, DCA and DGB with distances of 1.68, 2.15, and 2.98, respectively. The 3IXN glide score of atrazine was − 2.858, and the glide energy (− 20.976) also formed 2 hydrogen bonds (Pi–Pi stacking) interacting with 6 and 7 bases of DAB and DCB at distances of 2.40 and 2.48, respectively. Atrazine had a top ranking and good glide score. The dichlorvos (PubChem ID 3039) glide score was − 3.749 and the glide energy was − 20.764. Dichlorvos exhibited a good glide score and potentially interacted with pBR322 (Fig. 7), which formed 2 hydrogen bonds interacting with 7 and 19 bases of DGA and DGB with distances of 2.43 and 2.64, respectively. The endosulfan (PubChem ID 3224) glide score was − 4.218, and the glide energy was − 28.011; endosulfan exhibited a good glide score and potentially interacted with pBR322 (Fig. 8), which formed 1 hydrogen bond interacting with 7 bases of DGA with a distance of 1.94.

### DNA cleavage

**Figure 9:**
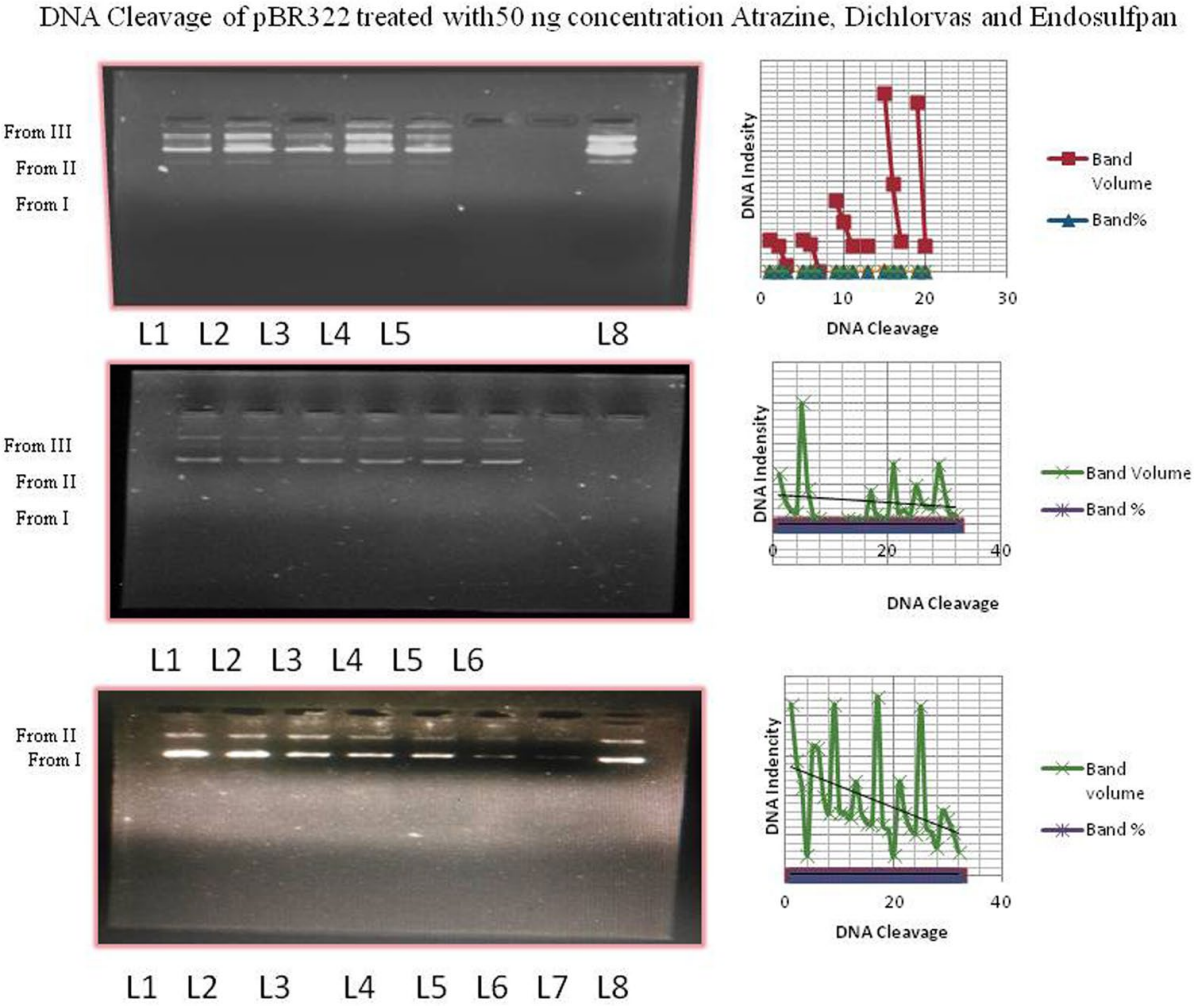
DNA cleavage of pBR322 treated with 50 ng/ml atrazine, dichlorvos and endosulfan.

The cleavage activity was demonstrated by gel-electrophoresis experiments using supercoiled (SC) plasmid pBR322 DNA in a medium TAE buffer. These experiments were monitored by the addition of varying concentrations of the pesticides (10–50 ng). When DNA was incubated with increasing concentrations of the pesticides, SC DNA was degraded to nicked circular form. The cleavage activities of pesticide 1 are depicted in Fig. 8. The activity of 1 starts at concentrations as low as 5 ng/ml, 10 ng/ml, 20 ng/ml, 30 ng/ml, 40 ng/ml, and 50 ng/ml, complete conversion of SC plasmid DNA into the NC form was observed, and the DNA completely smeared (Lanes 1, 2, and 3). In contrast, only 63% cleavage was achieved, and 37% cleavage was observed (Lanes 4, 5, and 6). To ensure that the pesticide was solely responsible for the cleavage, several control experiments were performed under identical conditions. When circular plasmid DNA is subjected to electrophoresis, relatively fast migration will be observed in the intact supercoiled form (Form-I). If one strand is nicked, the supercoiled DNA will relax and produce a slow-moving, open circular form (Form II). If all strands are cleaved, a linear form (Form III) is produced that migrates between Forms I and II. The cleavage effect upon irradiation of the plasmid pBR322 DNA in the presence of different concentrations of pesticide was tested. For the remaining two compounds, DNA cleavage is shown in Fig. 9. The form II content increases and the Form I content decreases gradually as the concentration of pesticides increases.

### Cell viability assay

**Figure 10:**
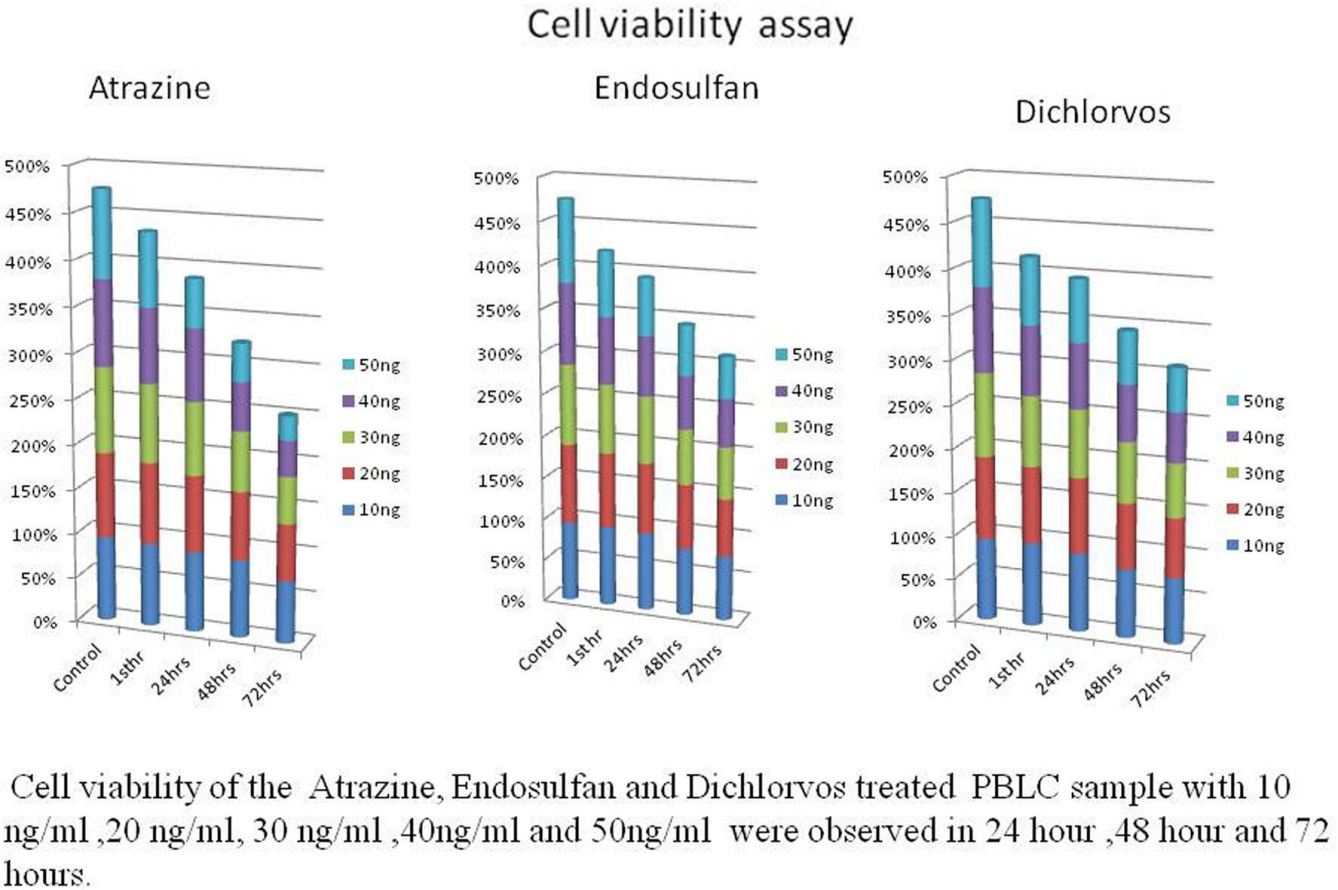
Cell viability assay. (Peripheral blood lymphocytes treated with different concentrations of 3 pesticides).

**Figure 11:**
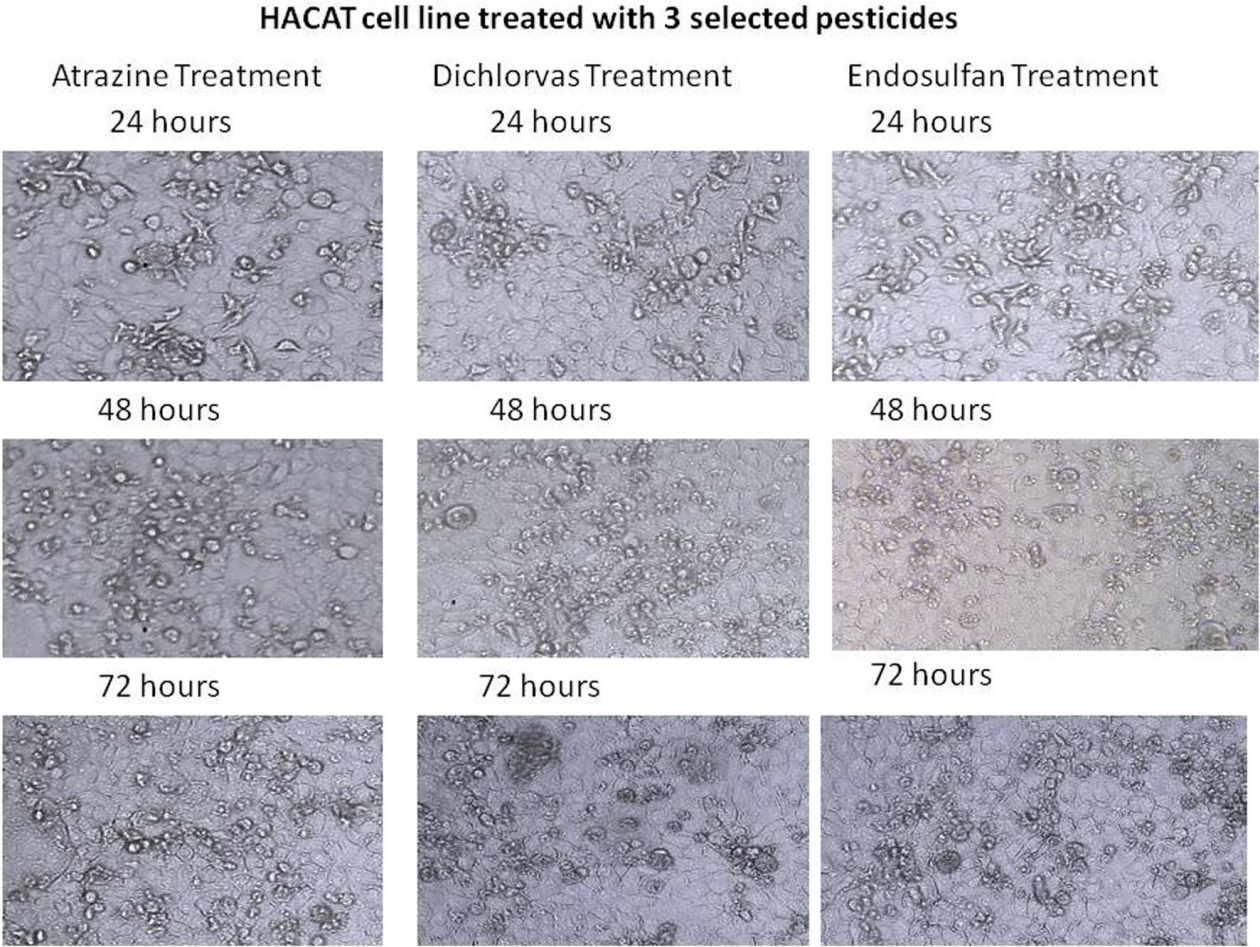
Cell viability assay (MTT assay). (HaCaT cell line treated with different concentrations of 3 pesticides).

**Figure 12:**
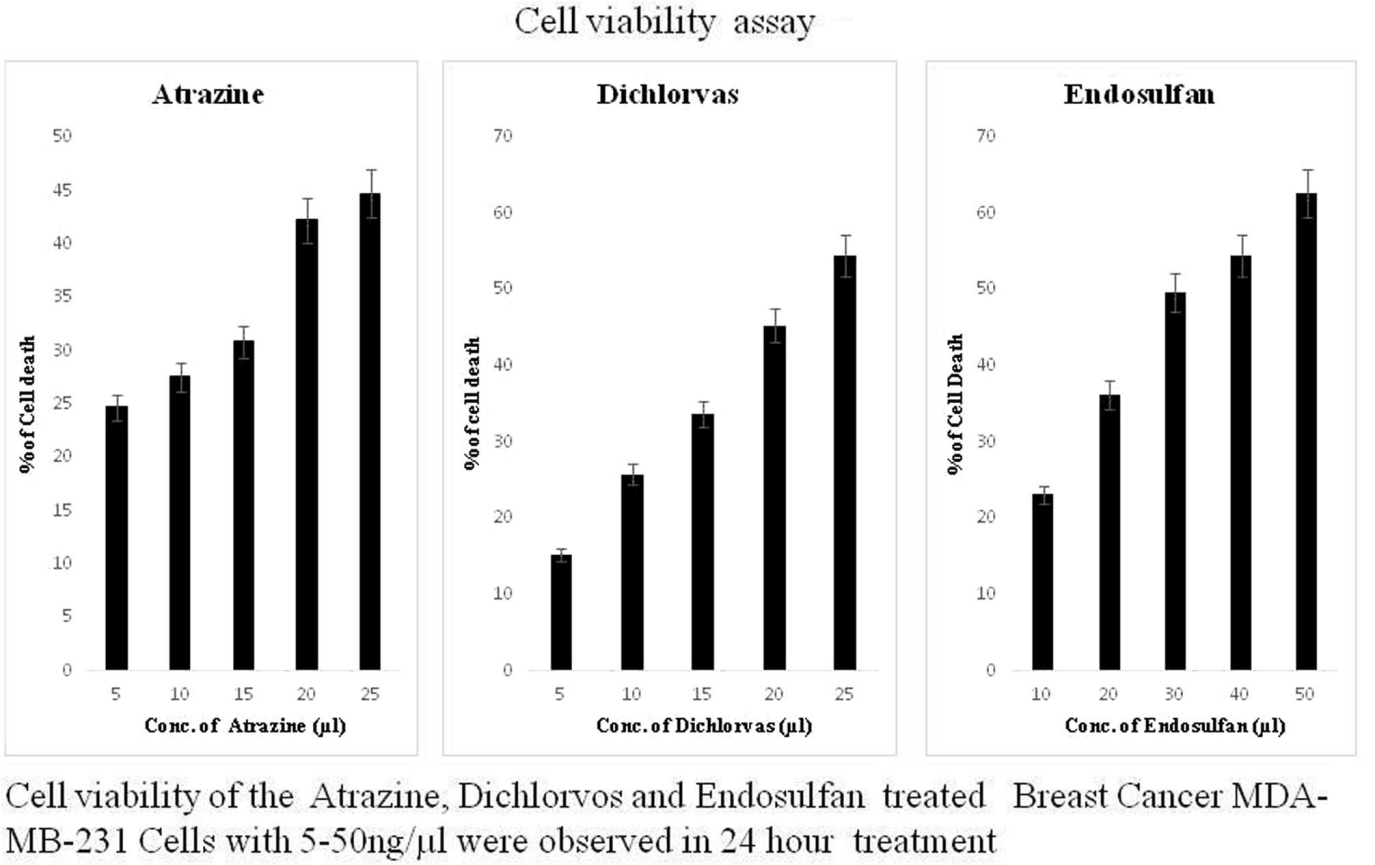
Cell viability assay (MTT assay). (MDA-MB-321 cell line treated with different concentrations of 3 pesticides).

Cell viability of the atrazine-, endosulfan- and dichlorvos-treated PBLC (peripheral blood lymphocyte cell) samples with 10 ng/ml, 20 ng/ml, 30 ng/ml, 40 ng/ml and 50 ng/ml treatment were observed at 24 h, 48 h and 72 h. The results showed a gradual decrease in cell viability as the time and concentration of the pesticide increased. The cell viability was lower in the cells treated with 50 ng/ml concentrations of the pesticides than other concentrations after 72 h of treatment. In the case of samples treated with 30 ng/ml pesticides, 75% of cells were viable after 72 h of incubation. In the case of 10 ng/ml pesticide treatment, 84% of the cells were alive. In the case of 40 ng/ml, viability gradually decreased after 72 h of treatment (Fig. 10). This trend shows that cell viability decreases with increasing concentration of pesticide and time of exposure. The results of this study clearly demonstrated that different types of pesticides were used to treat the HaCaT cell line, and cytotoxicity was determined using the MTT assay. Pesticide (atrazine, dichlorvos, and endosulfan) treatment resulted in increased toxicity in the HaCaT cell line, which was confirmed, based on the different treatment periods (24, 48, and 72 h) of the pesticides. HaCaT cells were treated with stranded concentrations of pesticides (atrazine, dichlorvos, endosulfan) for 24 h, 48 h, and 72 h. The death rates of HaCaT cells treated with atrazine, dichlorvos, or endosulfan were 28.2%, 21.7%, and 14.4%, respectively, after 72 h of treatment. The results of the MTT assay suggested that atrazine, dichlorvos, and endosulfan were toxic after treatment periods of 24, 48, and 72 h (Fig. 11). The MTT assay was performed on human breast cancer MDA-MB-231 cells after 24 h of exposure to 50 ng/µl concentrations (5–225 µl) of atrazine, endosulfan and dichlorvos. All three pesticides significantly inhibited the growth of cancer cells in a concentration-dependent manner, while the proliferation of untreated control cells remained uninhibited. The percentage of cell death increased remarkably with increasing concentrations of atrazine, endosulfan and dichlorvos, and the IC50 values were calculated using the calorimetric value of formazan production and was found to be 29.60 µl, 34.81 µl, and 22.69 µl, respectively. All experiments were performed in triplicate (Fig. 12).

### General, lifestyle and environmental factors associated with breast cancer risk

The findings reveal how the lifestyle patterns of women are affected among different age groups due to exposure to pesticides that increase their chances of developing breast cancer. The results showed that the 45–55-year age group had the highest risk, followed by the 55–65-year age group. When comparing the risk variables for married and unmarried women, married women had a high risk of BC. By classifying the risk of BC among educated and uneducated women exposed to pesticides in farmlands. For women who are mostly uneducated and are unaware of the harmful effects of pesticides, because of their lack of knowledge of pesticide exposure while using pesticides on agricultural lands, they were more likely to be exposed to pesticides than educated women. Table 4 shows data on general factors associated with breast cancer risk. The study on pesticide exposure is classified into eight different ways of pesticide exposure among women working on farmlands and other skilled workers on farmlands. The pesticide exposure levels are of $P_{1} ,P_{2} ,P_{3} ,P_{4} ,P_{5} ,P_{6} ,P_{7} \;and\,P_{8}$, which are defined as follows: $P_{1}$- working for '8' hrs on pesticide-treated farms, $P_{2}$- living nearby pesticide-treated farm areas, $P_{3}$-handling harmful pesticides, $P_{4}$-pesticides stored at home, $P_{5}$- consuming relatively high amounts of pesticide-treated food, $P_{6}$- washing of pesticide-sprayed clothes with bare hands, $P_{7}$- working with bare hands on pesticide-treated farms and $P_{8}$- no pesticide exposure. The results obtained based on pesticide exposure show that $P_{2}$ is higher than the other pesticide exposure levels, and people living near pesticide-sprayed farm areas had the highest risk of developing BC. Table 5 shows data on pesticide exposure and breast cancer risk. Our data show that the occupational status of women working in farmlands may be linked to an elevated risk of breast cancer.

**Table 4:** Age group Category used for analysis of breast cancer risk factors due to Pesticide exposure.

Age group	Pesticide exposure and mode of pesticide exposed groups															
Category	Patient								Control							
	P1 %	P2 %	P3 %	P4 %	P5 %	P6 %	P7 %	P8 %	P1 %	P2 %	P3 %	P4 %	P5 %	P6 %	P7 %	P8 %
> 25	–		–	–	–	–	–	42 (19)	–	1 (0.3)	–	–	–	–	–	49 (19.4)
25–35	–	6 (2.3)	–	1 (0.3)	–	–	–	1 (0.3)	-	2 (0.7)	–	–	–	–	–	24 (9.5)
35–45	3 (1.1)	9 (3.5)	–	2 (0.7)	2 (0.7)	3 (1.1)	-	4 (1.6)	-	1 (0.3)	–	–	1 (0.3)	–	1 (0.3)	39 (15.4)
45–55	7 (2.7)	30 (12)	1 (0.3)	–	4 (1.6)	2 (0.7)	1 (0.3)	2 (0.7)	4 (1.6)	–	–	3 (1.1)	1 (0.3)	–	2 (0.7)	10 (3.9)
55–65	2 (0.7)	5 (1.9)	–	3 (1.1)	9 (3.5)	1 (0.3)	12 (5)	4 (1.6)	–	1 (0.3)	3 (1.1)	7 (2.7)	1 (0.3)	–	–	9 (3.5)
≤ 65	6 (2.3)	28 (11.1)	3 (1.1)	2 (0.7)	15 (5.9)	8 (3.1)	19 (7.5)	3 (1.1)	2 (0.7)	5 (1.9)	8 (3.1)	2 (0.7)	6 (2.3)	8 (3.1)	3 (1.1)	12 (5)

(P1-$P_{1}$- Working before '8' hours of pesticide sprayed forms, $P_{2}$- Living nearby farm areas of pesticide application, $P_{3}$- Pesticides spraying women,$P_{4}$- Stored pesticides in their home, $P_{5}$- Consuming more amounts of pesticides applied vegetables,$P_{6}$- Washing work clothes worn during pesticides spraying, $P_{7}$- Farmers working with bare hands in pesticides applied forms and $P_{8}$- Without pesticide exposures).

**Table 5:** Lifestyle and general risk factors for breast cancer.

Risk factors	Percentage (%) Case	Percentage (%) Control
Age group (years)		
Below 25	0.9	0.5
25–35	3.96	11.1
35–45	19.0	24.2
45–55	37.7	32.4
55 and above	38.5	30.3
Tobacco users		
Chewer	32.9	5.8
Non-chewer	66.7	92.8
Marital status		
Married	98.0	95.2
Unmarried	1.6	4.8
Education		
Uneducated	33.3	35.7
Graduate	3.2	7.6
Higher secondary	6.0	14.0
High school & Intermediate	57.2	45.8
Occupation		
Farmer	49.2	40.1
Unskilled worker	4.4	14.5
Skilled worker	31.0	25.6
UN Employed	14.7	18.4
Food habits		
Veg Only	3.6	15.9
Veg and Non-Veg(Both)	96.0	82.6
Tobacco users		
Chewer	32.9	5.8
Non-chewer	66.7	92.8
Early menarche		
Yes (before the age of 12 years)	4.8	0.9
No (After 12 years)	94.8	99.0
Late menopause		
Yes (after the age of 55)	2.78	0.49
Before 55	97.2	98.5
Breast feeding		
Breast feeding given for 0–3 months	6.7	5.3
Breast feeding given for 3–6 months	21.8	16.4
Breast feeding given for 6–9 months	26.2	18.4
Breast feeding given for 1 year	11.9	10.1
Breast feed Not given	32.9	48.3
Abortion risk		
Miscarriage	44.9	7.7
No abortion	54.8	92.8
Associated cancer history with family members		
Frist degree relatives	7.9	–
Second degree relative	7.5	2.45
Third degree relative	11.1	0.4
Nil	73.0	97.0

## Discussion

The survey carried out by the Indian Council of Medical Research (ICMR) in metropolitan cities from 1982 to 2005 has shown that the incidence of breast cancer has almost doubled. Indian women with breast cancer are a decade younger than Western women, suggesting that breast cancer occurs at a younger premenopausal age in India, where environmental compounds and their exposure also increase cancer risk^21^. Both environmental and occupational factors have played a major role in increasing the incidence of breast cancer. Pesticides are the major threat of contamination because they are lipid soluble and nonbiodegradable, contaminating the environment and creating many adverse effects on human health, especially in inducing breast cancer. Pesticides containing harmful chemicals can induce cancer by altering hormones, causing DNA damage, tissue inflammation and gene silencing. Several studies have proven that pesticide usage affects the health of farmers. The present study concludes that farmers and unskilled workers are at a higher risk of developing BC than other skilled workers as a result of their frequent exposure to pesticides. Further studies are needed to confirm the role of pesticides in increasing the risk of breast cancer among women working in farmlands. Clinical studies have reported the link between breast cancer and increased blood sugar levels^22^. Studies have shown that an increased count of more than 11,000 white blood cells (leukocytes) per microliter of blood is associated with pesticide exposure, causing an increased breast cancer risk^23^. The measurement of intact pesticide residues or metabolites in blood can be an indicator to confirm immediate exposure to pesticides. Kabat et al. reported an increased level of WBCs due to pesticide exposure. Elevated levels of serum urea are positively associated with the risk of breast cancer in pesticide-exposed women. The experimental comet assay and chromosomal aberration analysis indicated that pesticides may cause carcinogenesis by damaging the integrity of DNA and the stability of the cell genome. Chromosomal aberrations cause an increased risk of breast cancer^24^. One of the causes is genotoxicity, and we discovered considerable DNA damage in pesticide-exposed people as a function of exposure periods in our subjects. This demonstrates that the length of exposure has an impact on DNA damage. The results of a study conducted by Shafiahammedkhan et al. reported abnormalities such as gaps and breaks, as well as a large increase in satellite associations in the chromosomal analysis of pesticide-exposed subjects compared to healthy individuals^25^. The experimental chromosomal aberration analysis indicated that pesticides may cause carcinogenesis by damaging the integrity of DNA and the stability of the cell genome. Chromosomal aberrations cause an increased risk of breast cancer. Karyotyping results of our studies indicated an increase in the length of the heterochromatin 1qh+ region and an increase in the satellite stalk of chromosomes 14pstk and 15pstk. These modifications may be due to pesticide exposure in the studied population. The molecular docking results revealed that pBR322 DNA showed good docking scores against the pesticides, and molecular dynamics confirmed the stability of the DNA-ligand binding. Our results further demonstrate the relationship between the chemical structure and biological function of pesticides. The docking scores between DNA and pesticides showed a strong binding affinity with a higher docking score. The top-ranking compound (atrazine) showed a good glide score, and a number of H-bonds and Pi-Pi stacking were observed. These docking studies revealed a significant binding affinity between pBR322 DNA and the selected pesticides. In silico docking studies show that all the compounds tested have the ability to interact with pBR322. The findings point to single-strand cleavage of supercoiled Form I to nicked Form II in a concentration-dependent manner. Under comparable experimental conditions, pesticide 1 (atrazine) exhibits more effective DNA cleavage activity than the other 2 pesticide compounds. Our results further demonstrate the relationship between the chemical structure and biological function of the pesticide. These docking studies revealed a significant binding affinity between pBR322 DNA and pesticide. The results of the cytotoxicity study (MTT assay) suggested that atrazine, dichlorvos, and endosulfan showed significant toxicity in nature after treatment periods of 24, 48, and 72 h in HaCaT and MDA-MB-231 cell lines. The comet assay results showed a gradual decrease in cell viability as the time and concentration of the pesticide increased. The cell viability was lower in the cells treated with 50 ng/ml concentrations of the pesticide after 72 h of treatment.

## Conclusion

Breast cancer has now become the most common cancer in most cities in India and the second most common cancer in rural areas. This may be due to the lack of awareness and early detection in developing countries such as India. Cancer is more common among the age group of 45 years and above. Late diagnosis leads to delayed disease presentations because of illiteracy and financial restrictions in some Indian areas, which in turn increase the mortality rate. India, a country relying on agriculture, has a large population of farmers who are exposed to pesticides during their course of work on farms. These pesticide-exposed communities become vulnerable to various disease conditions, among which cancer is the greatest risk. It is important for people to improve their knowledge on exposure to pesticides, prevention approaches, diagnosis and treatment. The role of pesticides as pathways to cancer development must be evaluated to address the current crisis of pesticide association with breast cancer. This increases the need for environmental monitoring and regulation. Therefore, the current study is a preliminary attempt to assess the age-based response to environmental carcinogens. A multidisciplinary approach will be required to investigate the risk factors associated with breast cancer along with traditional practices.

## Methods

### Study subjects

The study was conducted among South Indian subjects who were diagnosed with breast cancer during 2015–2019. It was approved by the Institutional Human Ethics Committee (IHEC) of Bharathiar University (Ref. No: BU/HGMB/S. S/HEC/2016/06) and Erode Cancer Center Hospital (Ref. No: ECR/319/Inst/TN/2013). The research guidelines of the Declaration of Helsinki framed by the World Medical Association (WMA) were strictly followed. Informed consent was obtained from the subjects and controls enrolled in this study. A total of 251 breast cancer subjects and 204 controls were recruited for this study. A detailed questionnaire was circulated among breast cancer and control samples to obtain relevant data required for this study. The first group comprised women below the age of 25, and their pesticide exposure period was calculated. The second constituted patients in the age group of 25–35 with an exposure period of 5 years. The third group contained patients between the ages of 35–45 years, and their pesticide exposure period was 10 years. The fourth group included patients aged 45–55 years who were exposed to pesticides for more than 15 years. The fifth group of patients was aged 55–65 years and 65 years and above, and they were all exposed to pesticides for more than 20 years.

### Sample collection

Peripheral venous blood samples were collected from the breast cancer subjects and controls aseptically using 3.0 ml EDTA and 2.0 ml heparinized tubes with disposable syringes. Samples were brought to the culture laboratory in sterile tightly covered ice-packed plastic containers to perform biochemical, chromosomal and genotypic analyses. The samples were stored at − 80 °C.

### Biochemical analysis

The blood samples were analyzed using an Erba GRA H360 Hematology Analyzer to determine the biochemical parameters of blood sugar, white blood cell count, hemoglobin, blood urea, platelet count and serum creatinine level.

### Analysis of DNA damage using single gel electrophoresis

DNA damage analysis (comet assay) was performed according to procedures previously described by Alok et al. 2013. HikaryoXLTM RPMI medium was used in this assay, and 0.75% normal melting point agarose was placed onto frosted slides with cover slips and left to gel for 10 min at 4 °C. Twenty microliters (20 ul) of lymphocytes was then added to 0.5% low-melting agarose (37 °C). After removing the coverslips, a second layer of sample mixture was pipetted onto the precoated slides and allowed to rest for 10 min at 4 °C. The coverslips were removed, and a third layer of 0.5% low-melting agarose was pipetted onto the slides, which were then allowed to gel for 10 min at 4 °C. The slides were immersed in a freshly produced cold lysis solution and chilled overnight. After that, the slides were placed in alkaline buffer for 20 min to allow the DNA to unwind. Electrophoresis was carried out for 25 min at a voltage of 0.66 V/cm and a current of 300 MA. The slides were then drained, placed on a tray, and washed 3 times for five minutes each with neutralizing buffer. DNA was precipitated, and slides were dehydrated for 10 min in absolute methanol before being dried at room temperature. To avoid artificial DNA damage, the entire procedure was carried out in low light. Ethidium bromide was used to stain the slides (25 m in PBS). DNA damage was performed using a 40 × objective with a fluorescence microscope.

### Study of chromosomal aberrations

Chromosomal abnormalities (karyotyping) were carried out according to the procedure reported by Hungerford et al.^26^. HikaryoXL™ RPMI Medium and colchicine from the Himedia laboratory were used in this study. Establishment of the culture: 2.0 ml of venous blood from breast cancer (approximately 30 drops) was inoculated into a culture vial containing 5.0 ml medium. The cultures were incubated at 37 °C for a period of 72 h and shaken periodically twice a day. The dividing cells were arrested at the meta-phase stage by adding 0.05 ml of colchicine solution (0.01%) at 30 min, and the contents of the vials were centrifuged at 1000 rpm for 20 min at the end of colchicine treatment. Six milliliters (6 ml) of prewarmed hypotonic solution was added to the pellet after disturbing the cell bottom. The contents of the test tubes were incubated for 7 min at room temperature. After incubation, 1 ml of freshly prepared fixative was added and centrifuged at 1000 rpm for 10 min. This step was repeated two or three times until a colorless pellet was obtained. A test slide was prepared by placing a drop of the cell suspension on a clean, chilled slide and dried immediately at 40 °C for a few seconds on a hot plate. The slide was examined under a microscope to see whether the concentration of cells and the spread of the chromosomes enabled detailed examination of meta-phases. A 4% Giemsa stain solution was used for staining. The GTG banding technique was executed using trypsin and Giemsa. The slides bearing chromosome spreads were treated with 0.25% trypsin for 3–10 s. This enabled the digestion of the cell membrane and cytoplasm so that the metaphase chromosomes could be analyzed as alternating light and dark bands. Chromosomal analysis showed fifty well-spread metaphase chromosomes were observed under the oil immersion lens of the light microscope, and selected metaphase chromosomes were photographed.

### Molecular docking

Molecular docking was performed using Maestro Glide version 11.8, Schrödinger 2018–4 modules from suite, and the process was performed by removing all the water molecules. Hydrogen bonds were added to the assignment, and the energy was minimized using the OPLS3e force field with exhaustive sampling. Eventually, the selected conformer was filtered for the minimization steps for a constant relative energy window of 10 kcal/mol with a minimum atom deviation of 1.00 Å. The inhibitory compounds used for docking were initially screened using standard precision (SP) docking followed by extra precision (XP) docking. Since XP mode is a refinement tool designed for use on only one inhibitory compound pose, large sets of compounds are screened by the SP mode. Finally, the prepared ligand structures were filtered and selected based on removing the most common repetitive structures. The scoring function is used to score the poses. The best docked structure was determined, and the compound was identified using the docking score function and Glide energy by the Glide XP Visualizer panel.

### DNA cleavage

DNA cleavage was observed, ruling out the possibility of DNA cleavage via the OH-based depurination pathway and possible oxidative cleavage. GeNei™ pBR322 (cesium chloride purified) was used in this study. DNA cleavage studies were conducted using supercoiled (200 ng) pBR322 DNA treated with 10 nanograms to 50 nanograms pesticide (atrazine, dichlorvos and endosulfan), and 5 mM Tris HCl/50 mm NaCl buffer (pH 7.2) was adjusted. DNA- and pesticide-containing vials were incubated for 2 h at 37 °C. A loading buffer and 30% glycerol were added, and electrophoresis was performed in TAE buffer at 50 v for 1 h using a 1.2% agarose gel containing 1.0 mg/ml ethidium bromide. The results were visualized using the Medicare gel documentation system, and the gel pictures were photographed.

### Cell viability assay

The dye exclusion test was used to determine the number of viable cells present in a cell suspension. This method is based on the idea that live cells have intact cell membranes that keep dyes such as trypan blue, eosin, and propidium out, whereas dead cells do not. In this test, dye is added to a cell solution, and the cells are visually examined to see if they take up or reject the dye. In this technique, a live cell will have a clear cytoplasm, while a nonviable cell possesses a blue cytoplasm. Himedia HikaryoXL™ RPMI Medium and Invitrogen Bioserves India Pvt. Ltd. Trypan blue was used. Countess cell counting chamber slides were used in this study. An aliquot of the cell suspension was centrifuged at 1000 rpm for 5 min, and the supernatant was discarded. The size of the aliquot depends on the approximate number of cells present. The aliquot should contain a convenient number of cells to count in an automated cell counter when suspended in 1 ml PBS and then diluted again by mixing with 0.4% trypan blue. The cell pellet in 1 ml PBS or serum-free complete medium is also used. One part of 0.4% trypan blue and 1-part of the cell suspension (dilution of cells) were mixed, and the mixture was incubated for 3 min at room temperature. Cells should be counted within 3 to 5 min of mixing with trypan blue, as longer incubation periods will lead to cell death and reduced viability counts. An Applied Biosystems automated cell counter was used to visualize live and dead cells.

### MTT assay

Cell Line: HaCaT and MDA-MB-231 cells were maintained in calcium-free Dulbecco’s modified Eagle's medium (DMEM) with 10% chelex-treated fetal bovine serum (FBS) and 1% penicillin‒streptomycin (Gibco- Lifetech, Karlsruhe, Germany). In the incuator, the cells were kept at 37 °C in a humidified atmosphere of 95% air and 5% CO_2_. The cell line was purchased from the National Center for Cell Science in Pune, India. The effect of atrazine, Dichlorvos, and endosulfan on the viability of HaCaT and MDA-MB-231 cells was determined by using 3-(4,5-dimethylthiazol-2-yl)-2,5-diphenyltetrazolium bromide (MTT). The MTT assay was performed as described in previous studies. Procedure and Cell Proliferation Assessments: Seed200 μl cell suspension in a 96-well plate at the required cell density (20,000 cells per well) without the test agent. The cells were allowed to grow overnight. A total of 1 × 10^4^ cells were seeded per well in a 96-well flat-bottomed microplate, and atrazine, dichlorvos, and endosulfan were applied for 24, 48, and 72 h to discuss the ability of HaCaT cells to inhibit cell proliferation. After incubation, the cells were washed twice with PBS, and 200 µl of culture medium containing 5 mg/ml MTT dye was added to each well and incubated further for 24, 48, and 72 h at 37 °C in a 5% CO_2_ atmosphere. After the incubation period, the plates were removed from the incubator, the spent mediawas removed, and MTT reagents were added to a final concentration of 0.5 mg/mL total volume. The plate was wrapped with aluminum foil to avoid exposure to light. Gentle stirring in a gyratory shaker enhanced dissolution. Occasionally, pipetting was required to completely dissolve the MTT formazan crystals, especially in dense cultures. The optical density (OD) was measured at 540 nm using a microplate reader (FLUOStar Omega—BMG Labtech) after the plates had been agitated for 10 min.

## General and lifestyle factors

The breast cancer patients’ general characteristics, lifestyle patterns and menstrual and pregnancy histories were statistically studied. The above results were statistically assessed using Statistical Package software (SPSS) in the recruited breast cancer patients.

## Supplementary Information

Supplementary Information.

## Data Availability

The datasets generated and/or analyzed during the current study are available from the corresponding author on reasonable request.
