# Supplementary material for: Predicting the relationship between pesticide genotoxicity and breast cancer risk in South Indian women in in vitro and in vivo experiments
Source: Sci Rep. 2023 Jun 15;13:9712. doi: 10.1038/s41598-023-35552-3 (PMC10272204; doi:10.1038/s41598-023-35552-3)
Supplement: Supplementary file 1 — Supplementary Information. [file 41598_2023_35552_MOESM1_ESM.docx]

**
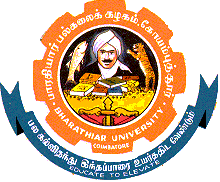
DEPARTMENT OF HUMAN GENETICS AND MOLECULAR BIOLOGY**

**MOLECULAR GENETICS AND CANCER BIOLOGY LABORATORY**

**BHARATHIAR UNIVERSITY**

**Research Title: “Environmental impact of Genetic Alterations in Women Breast Cancer Patients in South India”**

**bREAST CANCER QUESTIONNAIRE**

16BC008

**Sample Identification Number:**

Your answers to this questionnaire will be kept confidential and not be released to anyone, kindly help by completing this form as fully as possible. This will be extremely helpful to the doctors and Researchers.

Date:

Contact Number:

1. Name:
2. Date of birth: Year:
3. Sex:

1. Marital Status:
2. Height: cm Weight:
3. Education:

7. Food habits:

8. Occupation:

9. If employed, how long have you been working? :

10. Have you been regularly exposed to sources of pollution in your work Area? :

If yes, mention the source of pollution? :

11. Is your home near any agricultural land?:

If yes, mention the type of plantation:

12. Were pesticides ever used on crops grown on this farm ?:

If yes, for how long? :

13. Do you work on that land ?:

If yes, do you work within 24 hours of pesticide treatment? :

If yes, do you work with bare hands? :

14. Do you have any association of buying or transporting pesticides? :

15. Do you participate in the cleaning of the pesticide mixing or application equipment?:

16. Have you personally applied pesticides to any of the crops on the farm? :

If yes, which type of pesticides did you apply? Example:

17. Choose your mode of pesticide Treatment:

18. Does any of your family members work on a farm or agricultural area? :

If yes, what is your relation to them? : **-**

Do you have any contact with their working clothes, tools, and equipment with bare hands? :

19. Alcohol, Tobacco and Cigarettes product usage

| Product type | Chewed,  Smoked and  Drink | Not Chewed,  Smoke and drink | days/  week | Average daily consumption (quids/day/average) | How do you dispose the product? |
| --- | --- | --- | --- | --- | --- |
| Alcohol |  |  |  |  |  |
| Betel Nut |  |  |  |  |  |
| Smokeless(Chewing  Tobacco) |  |  |  |  |  |
| Light Cigarettes |  |  |  |  |  |
| Heavy Cigarettes |  |  |  |  |  |
| Others |  | | | | |

20. Medical history:

| Disease | Yes/No | How many days/years |
| --- | --- | --- |
| Anemia | Nil |  |
| Appendicitis operation | Nil |  |
| Asthma | Nil |  |
| Breast milky discharge | Nil |  |
| Breast soreness& Pain | Nil |  |
| Any Other Cancers | Nil |  |
| Diabetes Mellitus | Nil |  |
| Dizziness | Nil |  |
| Epilepsy | Nil |  |
| Heart disease | Nil |  |
| Hereditary problems | Nil |  |
| High blood pressure | Nil |  |
| Kidney Disease | Nil |  |
| Liver Disease | Nil |  |
| Ulcer | Nil |  |
| Others (Please specify) | Nil |  |

21. Type of Breast Cancer:

22. Breast Infected? :

23. Tests Undergone:

24. Initial Treatments:

26. Are you currently on any medication? :

27. Have you ever been sick in the past three years? :

28. Do you take medications on a regular basis (including prescription, over the counter, vitamins, herbals, etc.)?:

29. Are you allergic to any medication?:

30. Previous Radiation Therapy?:

31. Previous Chemotherapy?: Date of your last course:

32. Menstruation and Pregnancy History:

Age at Puberty:

When was your last period? :

Is your period regularly? :

If, yes, what is the number of days between periods? :

How many pregnancies (including abortion) have you had? :

Is there any complication before or after pregnancy? :

At what age is your first child? :

Have you breast feed your child? : ....If, Yes mentions the duration :

33. Family History:

Has anyone in your family been diagnosed with cancer? :

34. Do you currently have any of the following problems

Recent symptoms of:

35. Declaration:

I declare that all the preceding statements are true and complete to the best of my knowledge and belief. I accept to volunteer my blood sample for the research purpose.

| Signature | Date: |
| --- | --- |
